# Can knowledge of election results change recall of our predictions? Neural correlates of political hindsight bias

**DOI:** 10.1371/journal.pone.0220690

**Published:** 2019-10-10

**Authors:** Yin-Hua Chen, Hsu-Po Cheng, Yu-Wen Lu, Pei-Hong Lee, Georg Northoff, Nai-Shing Yen

**Affiliations:** 1 Research Center for Mind, Brain, and Learning, National Chengchi University, Taipei, Taiwan; 2 Mind, Brain Imaging and Neuroethics, Institute of Mental Health Research, Royal Ottawa Health Care Group, University of Ottawa, Ottawa, Ontario, Canada; 3 Graduate Institute of Humanities in Medicine, Taipei Medical University, Taipei, Taiwan; 4 Brain and Consciousness Research Center, Taipei Medical University, Shuang Ho Hospital, New Taipei City, Taiwan; 5 Center for Cognition and Brain Disorders (CBBD), Normal University, Hangzhou, China; 6 Department of Psychology, National Chengchi University, Taipei, Taiwan; UNSW Sydney, AUSTRALIA

## Abstract

Hindsight bias (HB) is the tendency to retrospectively exaggerate one’s foresight knowledge about the outcome of an event. Cognitive processes influenced by newly obtained outcome information are used to explain the HB phenomenon, but the neural correlates remain unknown. This study investigated HB in the context of election results using a memory design and functional magnetic resonance imaging for the first time. Participants were asked to predict and recall the percentage of votes obtained by (pairs of) candidates before and after an election. The results revealed that 88% of participants showed HB by recalling that their predictions were closer to the actual outcomes than they really were; and participants had HB for 38% of the events. The HB effect was associated with activation in the medial superior frontal gyrus and bilateral inferior frontal gyrus (IFG), which have been implicated in updating an old belief due to new information and is similar to the process of reconstruction bias. Furthermore, participants with a greater HB effect showed greater activation of the left IFG. In conclusion, we successfully observed the HB phenomenon in election results, and our imaging results suggested that the HB phenomenon might involve reconstruction bias.

## Introduction

Hindsight bias (HB) is the tendency to retrospectively exaggerate one’s foresight knowledge about the outcome of an event [1,2]. Previous behavioral studies have affirmed that HB is a pervasive and robust phenomenon [3,4,5,6,7,8], such as found in medical diagnoses [9], consumer satisfaction [10], juror’s decisions [11], athletic competitions [12], public policy [13], business startups [14], and election results [15,16,17,18,19,20,21,22].

Both cognitive and motivational factors have been proposed to account for HB; however, the empirical evidence for motivational factors is generally weak [3,4,5,8] Therefore, in this study, we focused on cognitive factors. According to Hawkins and Hastie [3], when people are asked their original judgment after knowing the outcome of an event, they might recollect their old judgment from episodic long-term memory. The outcome information would prevent them from correctly recollecting the old judgment either by destroying or disturbing the memory trace of the original judgment or by reducing its accessibility [1]. Therefore, HB due to recollection bias has been defined as the effect of outcome information on the direct-recall process [5].

Alternatively, it is possible that people reconstruct their old judgment by anchoring on the outcome and then adjusting to infer their old judgment. HB occurs when people are unable to make a perfect adjustment [23,24]. Updating and re-judging is the other class of reconstructive process, which proposes that people make a retrospective judgment by simply repeating the prior judgmental process. However, the contextual information that affects the old judgment changes or becomes irrelevant or incongruent with the outcome information; thus, HB occurs [24, 25]. Therefore, HB due to reconstruction bias has been defined as the effect of outcome information on the processes of anchoring on the current belief and adjustment or re-judgment [5,6,26]. There are also other cognitive process models [27], for example, specifically focus on either memory processes such as the model Selective Activation and Reconstructive Anchoring (SARA) [6] and Reconstruction After Feedback with Take the Best (RASFT) [22]. However, to the best of our knowledge, no study has investigated the underlying cognitive processes of the HB phenomenon at the neural level. Therefore, the aim of this study was to investigate the possible neural correlates of HB using functional magnetic resonance imaging (fMRI).

In terms of experimental design, previous behavioral studies have often used a hypothetical or memory design to investigate the HB phenomenon [28]. In the case of a hypothetical design, participants are provided with the outcome information and then asked how they would have predicted the outcome had they not been provided with the outcome information. Their post-event hypothesized judgments have been compared with judgments either made without outcome information in a within-participants designs or from different participants who do not receive outcome information in a between-participants designs [1,19]. In the case of a memory design, participants are asked to make predictive judgments and to recall them in hindsight after receiving outcome information. Predictive and recalled judgments are compared within participants in this design [24,29].

Both studies using hypothetical [14,19,20,21] and memory designs [15,17,22,30] investigated HB in elections. For example, Synodinos [21] used a between-participants hypothetical design and asked a group of university students to predict the percentage of votes for three candidates, along with their confidence level for the predictions and the estimates of winning probability of each candidate 1 day before the 1982 Hawaiian gubernatorial election. The data were compared with another group of students who were asked to make the same judgments in hindsight as if they had not known the election outcome 2 days after the election. The results were somewhat mixed with regard to the HB effect. Overall, the two groups did not show differences in their estimated percentage of votes; however, some evidence for an HB was observed in that the post-election group was more confident in their estimates than the pre-election group and their estimates about the winning probability were distorted toward the election outcome for one of the three candidates. Powell [20] reported the HB phenomenon in university students selectively for the judgments of percentage of votes, probability of winning, and knowledge of the candidates, as well as the confidence levels of the judgments for the 1984 US Presidential, Missouri Gubernatorial, and Missouri Lieutenant Gubernatorial elections. Students selectively recalled having assigned higher probabilities and percentage of votes to the actual winners, remembered having more confidence in the accuracy of the percentage of votes, and claimed to have had more knowledge of the candidates than they had before the elections. Such results were consistent in both the hypothetical and memory designs [20]. Leary [14] and Fischer and Budescu [19] also reported a small but significant HB effect in the hypothesized estimates of the percentage of votes in the 1980 US Presidential elections and the 1992 Israeli Knesset elections, respectively. Even though the HB phenomenon can be detected using the hypothetical design, we argue that it is actually difficult for participants to indicate their hypothesized pre-election estimates by ignoring the actual election results that they might have learned from news or any discussion from friends or people nearby.

Indeed, many election studies have demonstrated the HB phenomenon by applying the memory design. For example, Tykocinski [22] asked university students to assess the chances of winning for the three major candidates in the Israeli prime minister election, each on a separate scale ranging from zero to very high (0 to 10) before the election. After the election, the students were asked to reassess what the chances of winning the election were for each of the three candidates retroactively and in view of the actual election outcomes. The results revealed the HB phenomenon for all voters even though they did not vote for the winner [22]. Similarly, the HB phenomenon has also been reported when participants were asked to predict the percentage of votes for the political parties and to recall their predictions for the 1998 German parliament election and the 2000 Nordrhein-Westfalen state parliament election [15] as well as the 2002 German national parliament election [16]. Moreover, the HB phenomenon has also been reported when judging the winning probability of candidates using a memory design [30] and the percentage of votes of candidates with both designs [17] in the 2012 US Presidential election.

As no study in the existing literature has ever used fMRI to investigate the possible underlying cognitive processes of the HB phenomenon, especially using a memory design, we reviewed the studies that are conceptually similar to reconstruction bias; that is, information updating [31,32]. In information updating studies, people are asked to estimate the likelihood of life events and to indicate their estimates again after receiving information about the actual likelihood [31,32]. When participants updated their original beliefs, the left inferior frontal gyrus (IFG) and medial frontal cortex/superior frontal gyrus (MFC/SFG) tracked desirable estimation errors, whereas the right IFG tracked undesirable estimation errors [31].

In summary, we investigated the neural correlates of the HB phenomenon with a memory design using fMRI. Specifically, we investigated the HB phenomenon in the context of a political election event. Participants were asked to predict the percentage of votes received by candidates before an election and to recall their original prediction after knowing the election results. We expected to replicate the HB effect at the behavioral level as found in previous studies [15,17,22,30]. We also expected that the HB phenomenon would involve reconstruction bias. Thus, as an effect of information updating participants would show greater brain activation in their bilateral IFG and medial MFC/SFG. In addition to an exploratory whole-brain voxel-wise analysis, we also performed region of interest (ROI) analyses to test whether the aforementioned regions that have been previously implicated in information updating [31,32] would also respond to HB phenomenon. If so, we expected to observe the correlation between the corresponding brain activation intensity with the behavioral HB effect.

## Materials and methods

### Participants

The election investigated in this study was the 2014 Taiwan mayoral election that included six cities (i.e., Taipei, New Taipei, Taoyuan, Taichung, Tainan, and Kaohsiung). Thus, we recruited participants equally from the six cities as much as possible (see Table 1 for details of the participants’ residence). The mayoral election was an extremely important event in Taiwan; people generally follow politics closely by showing a high voting rate (66.52% for the 2014 mayoral election across the six cities). Moreover, participants were aware that the experiment was related to the mayoral election from a recruitment flyer for the experiment; thus, we believed that they could make decent judgments as required in the experiment. The participants only knew that they had to complete two sessions of the experiment before and after the election; they were unaware of the purpose and hypothesis of the study until they completed both sessions of the experiment. Twenty-six participants (16 females and 10 males; mean age = 26.19 ± 3.75 years) were recruited for the pre-election session (1–23 days before the election; see Table 1 for details), and 24 (15 females and 9 males; mean age = 26.42 ± 3.75 years) returned for the post-election session (37–69 after the election; see Table 1 for details). All participants were right-handed, with normal or corrected-to-normal vision, and without any neurological, psychiatric disorders, or contraindications to MRI. They signed written informed consent to participate in the study, and the procedures were approved by the Research Ethics Committee of National Taiwan University. All participants received a fee for completing each session of the experiment.

**Table 1:** Profile, behavioral data, and imaging analysis inclusion of participants.

Participant #	Gender	Residence	Age	Time interval before election (days)	Time interval after election (days)	Time interval between two sessions (days)	HB%	HB Size	Imaging analysis inclusion
**1**	F	Taipei	23	23	38	61	33%	8.5	Yes
**2**	M	Taipei	24	23	39	62	50%	5.33	Yes
**4**	F	Tainan	22	22	51	73	17%	10	Yes
**5**	F	Taoyuan	27	21	50	71	50%	5.67	Yes
**6**	F	Taoyuan	28	21	40	61	33%	9	Yes
**7**	F	Taichung	21	19	52	71	33%	7.5	Yes
**10**	F	Taichung	23	17	37	54	17%	10	Yes
**11**	M	Kaohsiung	28	17	36	53	0%	NA	No (all events without HB)
**12**	F	Taoyuan	24	16	45	61	67%	3.5	Yes
**13**	M	Taipei	29	9	51	60	33%	2	Yes
**14**	F	Taoyuan	26	15	43	58	67%	7.5	No (head motion)
**15**	F	Tainan	27	14	63	76	33%	10	Yes
**16**	F	Taipei	27	14	37	51	33%	3	Yes
**18**	F	New Taipei	24	9	51	60	50%	3	Yes
**19**	F	New Taipei	22	9	39	48	33%	6.5	Yes
**20**	M	Tainan	21	8	38	46	50%	6.67	Yes
**21**	M	Kaohsiung	30	8	44	52	50%	4.67	Yes
**22**	F	New Taipei	33	7	44	51	33%	7.5	Yes
**23**	F	Tainan	32	7	68	74	0%	NA	No (all events without HB)
**24**	M	New Taipei	31	6	40	46	100%	4.67	No (all events with HB)
**25**	M	Tainan	27	6	37	43	83%	10.8	Yes
**26**	M	Taichung	24	5	37	42	17%	30	Yes
**28**	M	Kaohsiung	27	4	36	40	0%	NA	No (all events without HB)
**31**	F	Tainan	34	1	45	46	33%	7	Yes
**Mean**			26.42	12.54	44.21	56.67	38.13%	7.75	
**SD**			3.75	6.74	8.51	10.76	24.80%	5.7	

### Materials, tasks, and procedures

#### 1. Pre-election session

In the pre-election session, participants were asked to write down their predictions for six pairs of candidates from six cities for the 2014 Taiwan mayoral election in a questionnaire. The reason for asking participants to provide their judgments for a pair of candidates for a given city (but not for a candidate for a given city) was because the candidates in each city were mostly from two major parties [i.e., Chinese Nationalist Party, also known as Kuomintang (KMT), and the Democratic Progressive Party (DPP) or pro-DPP] and the other parties rarely have a candidate that could compete with the candidates from the two major parties. We asked the participants to provide a “relative” percentage of votes for each pair of candidates that totaled 100% by ignoring the votes obtained by other candidates from other parties (Indeed, the resulting percentages of votes from the two major parties in the 2014 mayoral election were 97.98%, 98.84%, 98.97%, 100%, 100%, and 98.98% for Taipei, New Taipei, Taoyuan, Taichung, Tainan, and Kaohsiung, respectively).

#### 2. Post-election session

**Fig 1:**
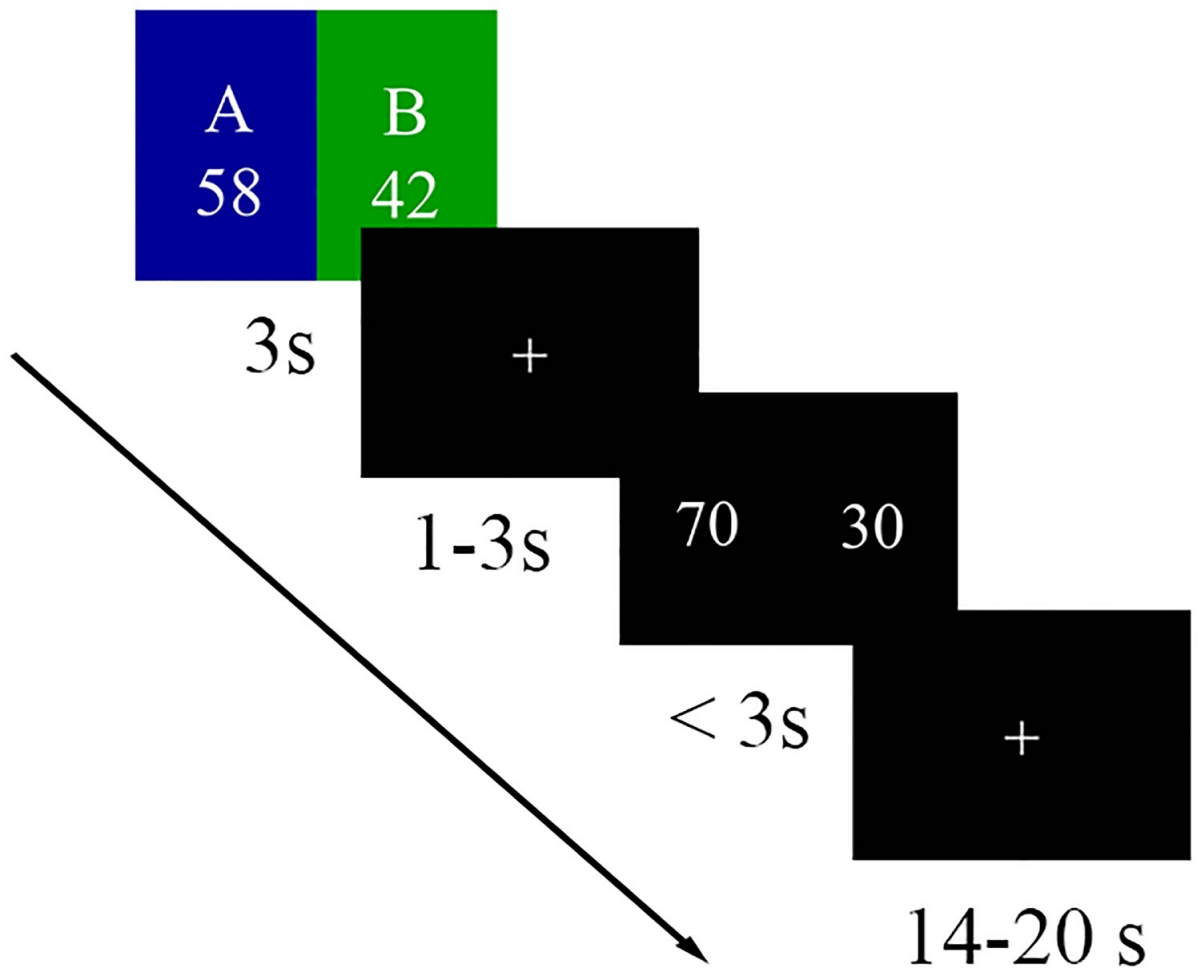
Timeline of an example trial during the post-election session. In each trial, the participants were provided with election outcome information for candidate A vs. B (58% vs. 42%) in a given city and they had to answer whether their recall of the prediction was similar to 70% vs. 30%.

Participants were asked to complete two tasks during the post-election session. The first task was a similarity judgment task while the subject underwent an fMRI scan. Participants were provided with the election outcome of a pair of candidates (the relative percentage of votes totaling 100%) and they had to answer whether their recall of the prediction was similar to one of nine different pairs of judgments (i.e., 30 vs. 70, 35 vs. 65, 40 vs. 60, 45 vs. 55, 50 vs. 50, 55 vs. 45, 60 vs. 40, 65 vs. 35, and 70 vs. 30). The nine different pairs of judgments were used to cover the possible judgments and to accumulate nine brain responses for each pair of candidates from each city. As shown in Fig 1, participants would first see the names of a pair of candidates for each trial, with the actual relative percentage of votes (totaling 100%) obtained by candidates under the name and with the political color of the political party (i.e., blue for KMT and green for DPP/pro-DPP) as the background color for 3 s. The side (i.e., left or right) assigned to KMT and DPP/pro-DPP candidates was randomized. During this 3 s, participants had to recall their prediction of the percentage of votes for this pair of candidates, followed by a fixation cross that appeared for 1–3 s (jittered). Next, a pair of vote percentages was presented, and participants had up to 3 s to judge whether it was similar to their prediction or not by pressing a key on the button box with their left or right thumb. The position (left vs. right) of the buttons referring to the responses (yes vs. no) was counterbalanced across participants. Finally, a fixation cross appeared for 14–20 s (jittered) as the inter-trial interval (ITI).

To rule out the effect of political color representing the parties (i.e., blue and green) shown in the background, the participants were asked to complete another ten trials in which they answered whether they liked the colors blue and green or not. Therefore, there were 64 trials completed. The trials were tested in random order. In addition to fMRI scans, participants also received an anatomical scan for 6 min. After completing the similarity judgment task inside the scanner, participants were asked to write down their exact recall of the predictions for the six pairs of candidates from the six cities (also the relative percentage of votes totaling 100%) in a questionnaire outside the scanner as a second task. The experiment took about 1 hr. The experimental program was written using MATLAB2008 (MathWorks Inc., Natick, MA, USA) and Psychotoolbox 2.5.4 was used to record the participants’ behavioral responses and brain images.

### Data acquisition

MRI images were collected with a 32-channel head coil in a 3T scanner (Skyra, Siemens Medical Solutions, Erlangen, Germany). A T2*-weighted gradient-echo echo planar imaging sequence was used for functional scanning, with a 3 mm slice thickness, 224 × 224 mm^2^ field of view, 90° flip angle, 35 slices, 2,000 ms repetition time (TR) and 30 ms echo time (TE). The anatomical, T1-weighted high-resolution image (1 × 1 × 1 mm^3^) was acquired using a standard MPRAGE sequence, with a 7° flip angle, 2530 ms TR, 3.3 ms TE, and 1,100 ms of inversion time.

### Data analysis

#### Behavioral data

We defined whether the participant had HB toward the given pair of candidates or not based on a proximity index suggested by Pohl [33] (see also 15,18). The proximity index is the absolute value of the difference between the original judgment and the actual result minus the absolute value of the difference between the recalled judgment and the actual result (where the recalled judgment was the value written down by participants in the final recall task of the post-election session). When a recalled judgment is closer to the actual result, the index is positive, namely there is HB. Otherwise, the recalled judgment could be farther away from the actual result, or was exactly the same as the original prediction showing a successful recall. For example, if a participant predicted the election outcome of a given pair of candidates (candidate A vs. candidate B) as 55% vs. 45%, the actual outcome was 65% vs. 35%, and the participant recalled the prediction as being 60% vs. 40%, then the proximity index was positive (i.e., for candidate A, the calculation was|55 − 65|−|60 − 65| = +5, which is the same for the candidate B, |45 − 35|−|40 − 35| = +5, as the relative votes totaled 100%), indicating that the participant had HB toward the given pair of candidates. We calculated the participant’s mean proximity index toward the six pairs of candidates (hereafter HB size) and tested whether it was significantly different from 0 using the one-sample *t*-test with an α-value of .05. Moreover, we calculated the percentage of participants that showed HB (i.e., who showed an HB size greater than zero) among the six pairs of candidates representing the six cities (hereafter HB%; for example, having HB toward one pair of candidates among the six pairs of candidates indicated a 17% of HB%). Both HB size and HB% were used as behavioral indices to correlate with the brain imaging data in the following region of interest (ROI) analysis (details see ROI analysis) to test whether there would be a significant brain-behavioral relationship. IBM SPSS 20.0 (IBM Corp., Armonk, NY, USA) was used for the statistical analysis. Effect sizes were calculated using Cohen’s *d*.

### Imaging data

#### Whole brain voxel-wise analysis

The imaging analysis was performed using the Statistical Parametric Mapping 8 (Wellcome Trust Center for Neuroimaging, London, UK) software package. The functional images of each participant were first corrected for slice timing and head motion and then co-registered to the participant’s segmented gray matter image. Next, the images were normalized to the standard Montreal Neurological Institute (MNI) template, and spatially smoothed by convolution with an 8-mm full width at half maximum Gaussian kernel. We modeled the individual data using a general linear model with up to seven regressors with the following brain images: when participants showed HB and did not show HB (hereafter non-HB) as they were presented with the names and election outcomes of the candidates and they were asked to recall their predictions (hereafter recall period) for the experimental trials (regressor 1 and regressor 2); when they were presented with the political color of the two parties in the color trials (regressor 3); when they were presented with the fixation cross in all of the trials (after the recall period in the experimental trials and after seeing the political color of the two parties in the color trials) (regressor 4); when they made a judgment in all of the trials (regressor 5); when they were presented with the fixation cross during the ITI in all trials (regressor 6); and when they did not respond within 3 s during error trials (regressor 7). Each regressor was then convolved with a canonical hemodynamic response function to model the expected blood-oxygenation-level-dependent signal. In addition, the six realignment parameters were included in the model to regress out potential movement artifacts. Specifically, regressor 1 and regressor 2 were regressors of interest to investigate the brain activation provoked by the HB effect, whereas the other regressors were not of interest. For the group level analysis, the parameter estimates between regressors 1 and regressor 2 (i.e., when participants showed HB contrasting to when they did not show HB during the recall period; hereafter “HB > non-HB” and “non-HB > HB”) of each participant were fed into a one-sample *t*-test using a random-effects analysis. The time interval between two sessions for each participant served as the covariate of non-interest to rule out the possible effect of retention interval [34]. The threshold of the statistical maps was set at a voxel-wise intensity of *p* < .005 (uncorrected) with a false discovery rate correction at the cluster level using the whole brain as the volume of interest. The resulting regions of activation were characterized in terms of their peak voxels in the MNI coordinate space.

#### ROI analysis

In addition to the whole brain voxel-wise analysis, we performed a ROI analysis in the regions following the literature on information updating [31]. We used the three regions reported [31] as ROIs to examine whether HB was associated with the mechanism of information updating, namely left IFG, right IFG, and MFC/SFG. We transferred the reported coordinates of the peaks of the three regions from Talairach to MNI coordinates in (−59, 21, −1), (49, 16, 11), and (−13, 63, 28), respectively, as centers of the spherical ROIs with a radius of 6 mm. For each hypothesis-driven ROI, we extracted the mean parameter estimates (i.e., beta weights) averaged across the whole ROI associated with the contrasts of HB > non-HB, above the threshold of voxel-wise intensity of *p* < .001 (uncorrected). Subsequently, correlational analyses between the parameter estimates of the ROIs and HB size as well as HB% were carried out using Pearson’s product-moment correlation tests with an α-value of .05.

## Results

### Behavioral correlates of HB

As shown in Table 1, 88% of the participants (i.e., 21 of 24) showed HB for at least one of the six pairs of candidates. On average, participants showed HB for 38% (SD = 25%) of the events. Moreover, their mean HB size was significantly greater than 0, *t*_(20)_ = 6.229, *p* < .001, *d* = 2.786 (mean, 7.75).

### Neural correlates of HB

Five participants were excluded from the brain imaging analysis: participant #24 showed HB for all events; participants #11, #23, and #28 did not show HB for any of the events; participant #14 had excessive head motion with overall translation > 3 mm. The remaining 19 participants showed similar behavioral results as the 24 participants. They showed HB for 39% of the events, and their mean HB size was also significantly greater than 0, *t*_(18)_ = 5.794, *p* < .001, *d* = 2.731 (mean, 7.928).

#### Whole brain voxel-wise analysis

**Fig 2:**
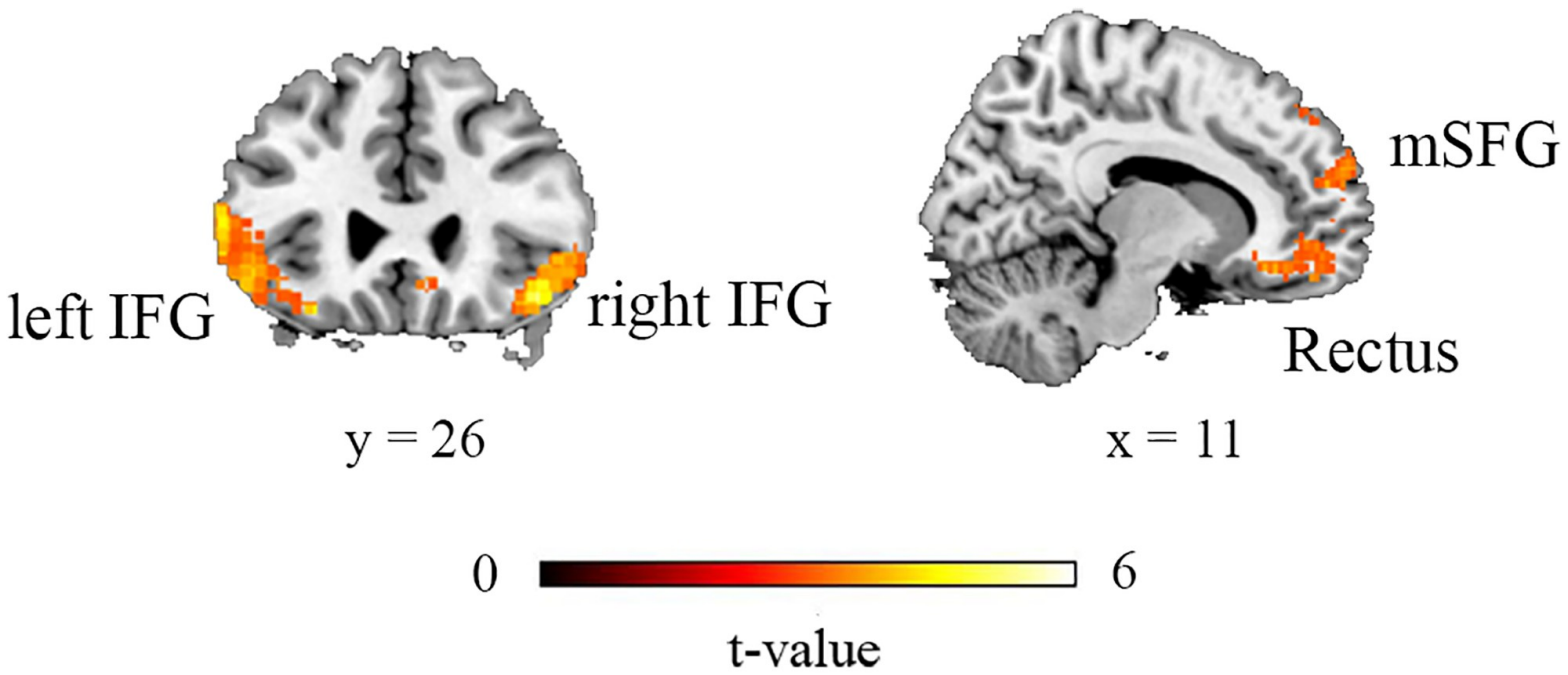
Brain activation associated with hindsight bias (HB). Significantly greater activation of clusters was induced when participants showed the HB phenomenon versus when they did not show the HB phenomenon after the election.

The participants showed higher activation in the medial part of the SFG, bilateral IFG, and rectus in the comparison of HB > non-HB (see Fig 2). The reverse comparison (non-HB > HB) did not reveal any significantly activated clusters.

#### ROI analysis

**Fig 3:**
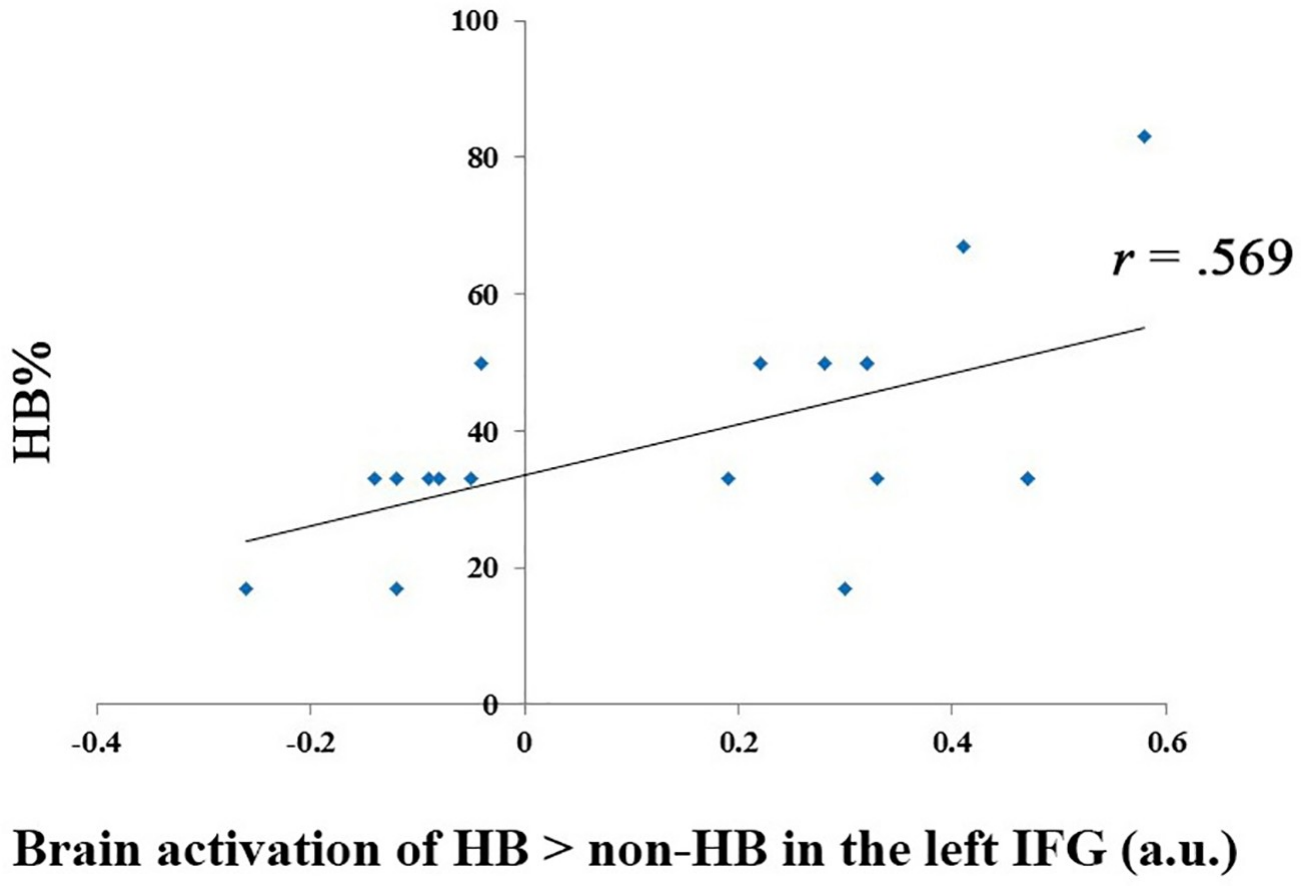
Correlation between percentage hindsight bias (HB%) and the left inferior frontal gyrus (IFG). Participants who had a greater number of events with HB phenomenon (i.e., HB%) showed greater activation intensity in the left IFG when participants showed the HB phenomenon versus when they did not show the HB phenomenon after the election.

A significant positive correlation was detected between HB% and the parameter estimates of the left IFG (i.e., ROI as reported [33]) in the comparison of HB > non-HB, *r*_19_ = .569, *p* < .05 (see Fig 3). No significant correlations were observed between other ROIs and the HB effect (i.e., HB% and HB size).

## Discussion

In this study, we successfully observed the HB effect in the context of political election results as in previous studies that used a memory design [15,17,22,30]. We found that 88% of participants showed evidence of the HB effect in at least one of the six tested conditions; across participants, we observed evidence for the HB effect in a sizeable proportion of the events (i.e., 38%), with a mean size of 7.75.

We report the neural correlates of the HB effect in the mSFG, the bilateral IFG, and the bilateral rectus gyrus for the first time. These findings could not be explained by a difference in memory retention [34] because we entered the time interval between pre-election and post-election sessions as a covariate in the model and regressed out its effect. As the mSFG and the bilateral IFG have been implicated in the mechanism of information updating [31], we also performed a ROI analysis of these three regions as reported previously [31] to investigate whether the mechanism of information updating is also implicated in HB. The activation level of the left IFG reflected the HB effect (i.e., HB%), reinforcing the plausibility of the component of reconstruction or updating one’s representation of the situation given new outcome information [3,5]. Our imaging results generally corresponded with previous findings that reconstruction bias represents a major source of the HB phenomenon [5].

Specifically, as found in the information updating studies that are conceptually similar to reconstruction bias, the left IFG has been associated with desirable estimation errors, such as when learning that the risk of experiencing future negative events like cancer is lower than the original prediction [31]. Interestingly, an enhanced response to bad news, but not a decrease in response to good news, was found in a follow-up study that used off-line repetitive transcranial magnetic stimulation to disrupt function of the left IFG [35]. The authors suggested that the left IFG inhibits updating in response to bad news, as the left IFG is thought to mediate different forms of inhibition, such as inhibition of unwanted memories [36] and inhibition of working memory to resolve interference from previous trials [37] [38]. This result suggested that the HB effect might result from ignoring undesirable information. Nevertheless, as the left IFG was associated with processing of desirable information or ignoring undesirable information, it might represent a motivational factor for HB to a certain extent; that is, to maintain self-respect or make others think that they are reliable [39, 8].

False memory, compared to true memory in the retrieval phase, engages greater brain activation within the prefrontal cortex (PFC), including the medial SFG, the ventral medial PFC/ventral ACC, the left precentral gyrus, the bilateral IFG, and the left inferior parietal lobe [40]. These areas greatly overlapped in the regions detected in our imaging results; however, the aforementioned areas were not evoked due to the processing of outcome results/new information, as in HB phenomenon. Therefore, we discounted the false memory effects that occurred during the HB phenomenon.

In conclusion, on top of the HB phenomenon found in the behavioral level as in the literature [15,16,17,18,19,20,21,22], we provided neural correlates of HB cognitive processes for the first time [3,5,24]. Our imaging results suggested that reconstruction bias due to newly obtained outcome information might be associated with the HB phenomenon.

### Limitations

In this study, we asked participants to predict and recall the relative percentage of votes toward a pair of candidates for six different cities, which might not be an easy task. We suggest that a future study should compare the pre- and post-election judgments toward a single candidate at a time. Moreover, more candidates create more events, which avoids cases in which participants show HB for all events or do not show HB for any of the events. In this way, it would be possible to compare brain activation of different phenomena, such as correct recall, the HB phenomenon, and the reverse HB phenomenon. Also, a general memory test as well as questions about political knowledge for candidates are suggested to investigate the possible effect of memory ability and political knowledge of candidates in HB in the election results [16,17,30]. Finally, we acknowledged that at this moment our results were unable to disentangle whether our participants used the strategy of anchoring and adjustment or re-judgment to reconstruct their predictions. And our results cannot be used to decompose the HB phenomenon into other cognitive processes, such as recollection bias. Future studies are needed to investigate these issues.

## Supporting information

S1 Dataset Behavioral data and extracted beta value of imaging data of each participant. (XLSX)

S2 Dataset Imaging data of each participant. (RAR)
